# Role of hormonal risk factors in HER2-positive breast carcinomas

**DOI:** 10.1038/sj.bjc.6600844

**Published:** 2003-04-01

**Authors:** A Balsari, P Casalini, R Bufalino, F Berrino, S Ménard

**Affiliations:** 1 Istituto Nazionale Tumori, Via Venezian 1, 20133 Milano, Italy and 2 Chair of Immunology, University of Milan, Via Venezian 1, 20133 Milano, Italy

**Keywords:** hormone factor risk, HER2, epidemiology

## Abstract

Examination of parity, age at menarche and at menopause by HER2 status in a large series of breast carcinomas showed a statistically significant increased-frequency of HER2-positive tumours in lower risk subgroups. The findings suggest a difference in the protective role of hormone-related risk factors between HER2-positive and -negative tumours.

Clinical and molecular biology findings have led to the identification of tumour overexpressing HER2 (HER2+) as a distinct subset (comprising 20–25%) of breast carcinomas (Slamon *et al*, 1989; Ross and Fletcher, 1998,^1999^; Ménard *et al*, 2002). This subgroup is characterised by a high aggressiveness (Ferrero-Pous *et al*, 2000) and responsiveness to chemotherapy (Ménard *et al*, 1999b). Since HER2-positivity has been associated with hormone independence due to the absence of expression of hormone receptors and unresponsiveness to tamoxifen (Pietras *et al*, 1995; Carlomagno *et al*, 1996), we hypothesised that hormonal risk factors may not influence HER2-positive tumours. If this is the case, the proportion of HER2-positive tumours as part of all tumours should be higher in the protected subgroup than in the unprotected ones.

To test this hypothesis, in a large surgical database of breast carcinomas at the National Cancer Institute of Milan from 1968 to 1979, we analysed the frequency of HER2-positive tumours, determined by immunohistochemistry, according to parity and age at menarche and at menopause.

## PATIENTS AND METHODS

Two series of consecutive patients treated at the Istituto Nazionale Tumori in Milan, Italy, for primary breast carcinoma were considered: first, 1211 patients who underwent surgery in 1968–1969 and received no further treatment (Rilke *et al*, 1991) and second, 717 patients operated in 1978–1979 and who then received adjuvant chemotherapy (Ménard *et al*, 1999a). The two series were very similar as concerned age at diagnosis, age at menarche, age at menopause, percent of premenopausal cases, number of children, nulliparous cases (Table 1

**Table 1:** Characteristics of the two cohorts included in the analysis

	**First series**		**Second series**	
**Parameter**	**1211 patients**		**717 patients**	
Mean age at diagnosis (range)	55 (21–81)	1211^a^	53 (25–84)	717^a^
Mean age at menarche (range)	13 (10–20)	1002^a^	13 (7–19)	690^a^
Mean age at natural menopause (range)	49 (30–62)	649^a^	48 (27–77)	401^a^
Frequency of premenopausal cases	39%	1211^a^	43%	717^a^
Mean number of children (range)	1.6 (0–8)	970^a^	1.8 (0–11)	685^a^
Frequency of nulliparous cases	22%	970^a^	22%	685^a^
Frequency of HER2-positivity	23%	1211^a^	23%	717^a^

a Number of patients considered for series.

). Only natural menopause was considered. Immunocytochemical staining was retrospectively carried out on Bouin-fixed, paraffin-embedded tissue using a polyclonal antibody against HER2-specific peptide (kindly provided by DJ Slamon), for the first series and anti-HER2 CB11 (1 : 10 dilution, Ylem, Avezzano, AQ, Italy) for the second series. Both antibodies revealed a 23% of HER2-positivity and an overlapping staining on consecutive slides stained with these two reagents (Mezzelani *et al*, 1999).

The proportion of HER2-positive tumours was analysed, according to parity (1655 cases) and age at menarche (1692 cases) and at menopause (1050 cases). The subgroup with 0 or 1 child, and the subgroups of <12 or <45, respectively, for age of menarche and age of menopause were considered as control group (OR=1). The expected proportions (EP) of HER2-positivity were calculated assuming that HER2-positive tumours are not affected by hormonal risk factors and using the mean odds ratios (OR) reported for parity, ages at menarche and menopause (Brinton *et al*, 1988; Vatten and Kvinnsland, 1992; Kelsey *et al*, 1993).

Expected proportions were defined as HER2-positive frequency of control group divided by mean OR reported in the literature for the risk group considered. Differences in proportions were analysed using the *χ*^2^ test.

## RESULTS

The first step in the analysis considered the effect of parity on HER2-positive breast cancer risk. The proportion of HER2-positive tumours was found to vary from 20.9% in the group with zero or one child, to 23.8% in the group with two or three children, up to 30.5% for women with more than three children (Table 2

**Table 2:** Frequency of HER2-positivity in primary breast carcinomas according to parity, ages at menarche and menopause of the patients

**Groups**	**No. of cases**	**No. of HER2+ cases**	**Observed % HER2+**	**OR Mean from literature**	**Expected % HER2+ considering no protection for HER2+ tumours**
*Parity*					
0–1	805	168	20.9	1	20.9
2–3	709	169	23.8*	0.95	22.0
>3	141	43	30.5*	0.75	27.9
					
*Menarche*					
<12	247	53	21.5	1	21.5
12–13	806	184	22.8	—	
>13	639	158	24.7	0.85	25.2
					
*Menopause*					
<45	194	45	23.2	1	23.2
45–49	298	68	22.8	—	
>49	558	120	21.5	1.2	19.3
*P=0.03 by *χ*^2^. OR=Odds ratio.					

). The significant increase of frequency (*P*=0.03, *χ*^2^-test) of HER2-positive tumours, according to the parity, suggests that this factor protects only the HER2-negative tumours. Accordingly, assuming protection only for the HER2-negative tumour subset and using the ORs as described in Patients and Methods for parity, the expected proportions of HER2-positivity would be of 20.9, 22.0 and 27.9% in the respective groups, that is, quite similar to the observed frequencies (Table 2).

The two other hormonal risk factors for breast carcinomas recorded in our database, that is, ages at menarche and menopause, also appear to have a protective impact only on HER2-negative tumours. In fact, an increased HER2-positive tumour proportion was observed with increased age at menarche, which is associated with decreased risk, and a decrease in HER2-positivity was observed with increased age at menopause, which is associated with increased risk (Table 2). Again using the estimated ORs and assuming protection only for HER2-negative tumours, the expected frequencies of HER2-positivity are in the range of the observed ones (Table 2).

## DISCUSSION

Altogether our analyses suggest that the three hormone-related risk factors analysed seem to protect only HER2-negative tumour subset. These results may be interpreted also as opposite to a promoting effect of hormone-related risk factors for the HER2-positive subgroup. Indeed, some clinical data (Carlomagno *et al*, 1996) have suggested a detrimental effect of tamoxifen treatment in patients with HER2-positive tumours.

To date, the majority of risk factors for breast carcinomas identified are related to hormones. Identification of other risk factors, specific for the onset of HER2-positive tumours might be hampered by a clouding effect of HER2-negative tumours, which represent the great majority of breast carcinomas. Indeed, as long as HER2-positive tumours, which represent only 25% of cases and therefore of little weight among all breast carcinomas, are analysed together with the negative ones, it will be difficult to sort out risk factors for HER2-positive tumours.

In conclusion, our study suggests that HER2-negative and -positive tumour subsets are influenced differently by breast carcinoma hormone-related risk factors. To confirm these data, a cohort study specifically addressing the role of hormone risk-factors for breast cancer with respect to HER2 status is going on.
